# Phytochemical Analysis and Bioactive Properties of *Opuntia dillenii* Flower Extracts, Compound, and Essential Oil

**DOI:** 10.1155/2024/6131664

**Published:** 2024-09-14

**Authors:** Mohammad Elouazkiti, Manal Zefzoufi, Houda Elyacoubi, Chemseddoha Gadhi, Hafida Bouamama, Atmane Rochdi

**Affiliations:** ^1^ Department of Biology Ibn Tofail University Faculty of Sciences, Kenitra, Morocco; ^2^ Department of Biology Cadi Ayyad University, Marrakesh, Morocco

**Keywords:** essential oil, flowers, HPLC, isolated compounds, MS/MS, *Opuntia dillenii*

## Abstract

New research is exploring the enhanced efficacy of antioxidant and antimicrobial compounds developed from *Opuntia dillenii* flowers, a multifaceted source with pharmacological effects such as antioxidant and microbicide activity indexes showing diverse medical capabilities. The purpose of this study was to determine the chemical composition, isolate the active compounds, and evaluate their antioxidant properties as well as antibacterial potential through HPLC-MS in flower extract from *Opuntia dillenii*. The extracts were analyzed by high-performance liquid chromatography (HPLC), and essential oil compounds were identified by gas chromatography (GC). Antioxidant properties were assessed using DPPH and ferric-reducing power (FRAP) assays. Antibacterial potential was evaluated using disk diffusion and microdilution methods. Nutritional studies of the flower indicated that it contained moderate levels of sugars (4.27% ± 0.240), proteins (1.913% ± 0.268), and microelements (potassium as a major element), sodium, and calcium, with concentration values of 2.267%, 0.55%, and 0.424%, respectively. Total phenolic content ranged from 1.61 ± 0.37 mg GAE/g (hexane extract) to 34.45 ± 0.42 mg GAE/g (ethanol extract). The study highlighted the richness of secondary metabolites, such as methylated flavonoids (quercetin 3-O-rutinoside, isorhamnetin-3-O-rutinoside, and isorhamnetin-3-O-glucoside), and identified essential oil compounds like trimethylsilyl hexadecenoate, squalene, gamma-eudesmol, and citronellol. Antioxidant activities revealed stronger activity in the butanolic extract, while isorhamnetin-3-O-rutinoside exhibited moderate antioxidant effects. These results provide the rationale for the potential incorporation of *Opuntia dillenii* flower extracts in food, cosmetics, and pharmaceutical products as a sustainable natural alternative with broad implications for human health.

## 1. Introduction

*Opuntia* is an important forage resource that serves as a source of water and energy for animals [1]. *Opuntia dillenii* flowers are an important source of therapeutic agents due to their diverse pharmacological properties and chemical structures. The literature provides limited data regarding the phytochemicals and biological activities of *Opuntia dillenii* flowers. Flower extracts have various pharmacological activities, including antimicrobial and antioxidant effects [2], as well as anti-inflammatory effects [3, 4]. *Opuntia* is recognized for its various health benefits and medicinal properties, including anti-inflammatory activity that can be beneficial in reducing inflammation in the body and is often associated with various health conditions. In sub-Saharan traditional medicine, *Opuntia* flowers and fruits are used as antiulcerogenic and antidiarrheal agents. This suggests that they may help prevent or treat ulcers and diarrhea. *Opuntia* flowers are administered orally as an antihemorrhoid medication, indicating potential effectiveness in managing or relieving hemorrhoids [5]. The fruits and flowers of *Opuntia* are valued for their antioxidant content. They can serve as a promising source of natural antioxidants in various applications due to their high nutritional value [6]. Numerous studies have demonstrated the antiradical activity of polyphenols extracted from the fruits, seeds, flowers, and cladodes of *Opuntia* spp. [7, 8]. *Opuntia* is recognized for its abundant bioactive compounds that contribute significantly to its nutritional and potential therapeutic benefits. Among its notable components are betalains, including betaine, known for their potent antioxidant properties that combat oxidative stress by neutralizing free radicals in the body. In addition to betalains, *Opuntia* provides essential vitamins and minerals such as potassium, magnesium, and calcium. The seeds of this plant are rich in linoleic acid, an essential fatty acid, and contain phytosterols and polysaccharides composed of arabinose and galactose. Phenolic compounds like gallic acid, vanillic acid, ethyl 3,4-dihydroxybenzoate, ferulic acid, sinapic acid, and p-coumaric acid have been identified in its fruits and cladodes [9]. Furthermore, the petals of *Opuntia* contain compounds such as isorhamnetin 3-O-glucoside, kaempferol 3-O-arabinoside, isorhamnetin 3-O-rutinoside, and quercetin 3-O-glucoside [3], adding to its diverse array of bioactive substances. Understanding the variety and richness of these bioactive compounds in *Opuntia stricta* provides promising insights into its nutritional value and potential health benefits, including antioxidant and anti-inflammatory properties [10]. The nutritional characteristics and human health benefits of *Opuntia dillenii* contribute to its increasing economic importance. However, *Opuntia* flowers have received limited study due to the difficulty in obtaining them and their limited flowering time. *Opuntia* flowers are currently attracting a lot of attention due to their high levels of phytochemical and biological activities. These flowers may contain beneficial phytochemical compounds and have interesting biological properties.

The study primarily focuses on analyzing the components of essential oils and purifying the derivatives of the extracts. It also encompasses a comprehensive analysis of the chemical composition of *Opuntia dillenii* flower extracts, their biological activity, as well as methods for valorizing these flowers, highlighting their potential in various fields such as health, cosmetics, and food. The present study is aimed at evaluating the chemical profile through high-performance liquid chromatography (HPLC)–MS analysis, determining and comparing the polyphenol and flavonoid contents, assessing antioxidant properties using 2,2′-diphenyl-1-picrylhydrazyl(DPPH) and ferric-reducing power (FRAP) assays, and conducting antibacterial activity tests against four strains (two gram-negative bacteria: *Escherichia coli* and *Pseudomonas aeruginosa* and two gram-positive bacteria: *Staphylococcus aureus* and *Enterococcus hirae*) of *Opuntia dillenii* flower extracts and the isolated compound.

## 2. Material and Methods

### 2.1. Microorganism Material

The microbiological material used in this study consists of four strains, as follows: two gram-negative bacteria, *Pseudomonas aeruginosa* (CIP A22) and *Escherichia coli* (CIP 54.8); two gram-positive bacteria, *Enterococcus hirae* (CIP 5855) and *Staphylococcus aureus* (CIP 53154). All these microorganisms were sourced from the Pasteur Institute. They were regularly subcultured and stored at 4°C before being used in the study.

### 2.2. Plant Material and Preparation of Extracts

Fruits and flowers of *O. dillenii* were harvested in the Essaouira region at Had draa 31.5783°, longitude: −9.539° and altitude of 180 (m). The climate is semiarid Mediterranean. Dr. A. Ouhammou from the Faculty of Sciences Semlalia, Cadi Ayyad University, identified the plant material. A sample was deposited in the MARK Herbarium - UCA - FSSM (Herbarium Marc “13103”). The collected *Opuntia dillenii* flowers were dried, and the extraction process was carried out by maceration at room temperature for 4 days using a 70% ethanol/water solution. The resulting extract was further subjected to liquid–liquid extraction using solvents of increasing polarities, including hexane, dichloromethane, ethyl acetate, and butanol, until all-natural substances were exhausted. After each extraction, the solution obtained was filtered and then concentrated under a rotary evaporator at 45°C.

### 2.3. Extraction of Essential Oil

*Opuntia dillenii* flowers were air-dried at room temperature. Essential oils were obtained by steam distillation of plant material using a steam distillation setup. The hydrosol obtained after steam distillation was separated in the presence of hexane. Sodium sulfate was added to remove any remaining water traces, and then the solvent was evaporated using a rotary evaporator at 40°C. Once the solvent had evaporated, the resulting essential oil was stored in a dark place at 4°C until further use. The extracted essential oils were then subjected to GC-MS analysis and evaluated for their biological activity.

### 2.4. Nutritional Quality

Moisture was determined in three replicates by desiccation at 40°C to a constant weight, in accordance with the method described by the Association of Official Analytical Chemists [11]. Ash content was determined through three separate ashing procedures on the residue obtained after moisture determination at 600°C for 6 h [12]. The residue from the ash determination was dissolved in a 1/3 (v/v) HCl solution, heated for 1 min, and then analyzed for mineral constituents (calcium, sodium, and potassium) using an atomic absorption spectrophotometer. Suitable dilutions were made for each mineral to ensure precise readings, and the results were expressed in mg/100 g of dry matter. The total sugars were assayed according to the method described by Dubois et al. [13]. Protein content was estimated using the Bradford assay, which is based on the adsorption of the Coomassie blue dye G_250_ [14]. The pH was determined using a pH meter, and the acidity level of the studied extracts was determined using a pH meter to measure the initial pH of the solution. Subsequently, the acidity was determined by titration with 0.1 N NaOH following the AOAC method [11]. During titration, the volume of NaOH required to reach the endpoint, indicated by a change in pH, was recorded. The results were then expressed in grams of citric acid equivalent per 100 g of dry matter.

 $$\begin{array}{c} \text{Acidity}=\frac{\left(N\times V\times \text{meq of citric acid}\right)}{W} \end{array}$$

*N* represents the concentration of NaOH (0.1 N), *V* denotes the volume of NaOH used for the titration (in milliliters), *W* signifies the weight of the sample (in grams), and meq of citric acid is the milliequivalent weight of citric acid (0.064).

### 2.5. Polyphenolic Analysis

#### 2.5.1. Determination of Flavonoid Contents and Total Phenolic

The total phenolic and flavonoid contents were determined using the Folin-Ciocalteu method as described by Singleton, Orthopher, and Lamuela-Raventos [15] and the aluminum chloride method as described by Bahorun et al. [16], respectively. The total flavonoid content is expressed as mg rutin per g of dry extract weight, and the total phenolic content is expressed as mg of GAE per g of dry extract weight.

#### 2.5.2. Isolation and Purification

The butanol extract (3.08 g) was subjected to fractionation by chromatography on a silica gel column. The elution was performed using a mixture of CH_2_Cl_2_ (dichloromethane) and MetOH (methanol) with a gradual increase in the percentage of MetOH. The eluted fractions were combined based on the results of thin-layer chromatography (TLC), resulting in several fractions. Fraction 7 (1.1 g) eluted with CH_2_Cl_2_: MetOH (90 : 10) yielded a pure compound identified as Compound 1. The purity of Compound 1 was verified by TLC, and its identity was confirmed through further analysis using HPLC and nuclear magnetic resonance (NMR) spectroscopy. The NMR spectroscopy experiment on the pure compound was conducted using a spectrometer operating at 600 MHz for ^1^H NMR (proton NMR) and 150 MHz for ^13^C NMR (carbon-13 NMR). The solvent used for this analysis was dimethyl sulfoxide (DMSO) or deuterium oxide (D2O).

#### 2.5.3. HPLC-DAD-ESI/MS Analysis

Identification of the phenolic compounds of *Opuntia dillenii* flower extracts was carried out by HPLC-DAD-ESI/MS analysis performed on a Dionex Ultimate 3000 Chromatography System (CA, USA), which was equipped with a quaternary pump (HPG 3400 RS), an autosampler (WPS 3000 TSL), and a column oven (TCC 3000). For this method, a Kinetex C18 reverse-phase column (250 × 4.6 mm, 2.6 *μ*m particles) supplied by Thermo Fisher Scientific was utilized. The gradient separation involved solvent A (0.1% formic acid in water) and solvent B (100% methanol) according to the following program: 0–3 min, linear gradient from 5% to 25% B; 3–6 min, 25% B; 6–9 min, 25-37% B; 9–13 min, at 37% B; 13–18 min, 37-54% B; 18–22 min, 54% B; 22–26 min, 54-95% B; 26–29 min, 95% B; 29–29.15 min, return to initial conditions at 5% B; and from 29.15 to 36 min, at 5% B. The flow rate of the mobile phase was maintained at 1 ml/min. The injection volume was 10 *μ*l, and the peaks were detected at 280 nm. Mass spectrometry was performed using a TSQ Endura-type triple quadrupole (Thermo Fisher Scientific) equipped with a heated electrospray-type ionization source (H-ESI) in negative mode. The parameters were set as follows: the temperature of the mass vaporizer, the temperature of the ion transfer tube, and the electrospray voltage were set to −2.5 kV. The full scan MS acquisition mode ranged between m/z 50–1000 in Quadrupole 1 with a mass resolution of 0.7 m/z and a scan time of 0.5 s [17].

### 2.6. Determination of Antioxidant Activity

#### 2.6.1. DPPH-Free Radical-Scavenging Assay

The measurement of antiradical activity on *Opuntia dillenii* extracts and essential oils was performed by the DPPH free radical scavenging test using the procedure described by Sanchez-Moreno [18]. In fact, reaction mixtures containing 100 *μ*l of each extract at different concentrations were mixed with 900 ml of solution DPPH radicals (4 mg/100 ml methanol). The mixture was stirred and allowed to stand for 30 min at room temperature in the dark. Quercetin, a specific compound, was used as the reference compound. The absorbance of the mixture was measured using a spectrophotometer at 517 nm. The DPPH is a purple-colored chemical compound that turns pale yellow or colorless when reduced by an antioxidant. When a sample containing antioxidant compounds is added to a solution of DPPH, these antioxidants react with the DPPH, thereby neutralizing its oxidative effects. This reaction results in a change in the solution's color, which can be measured spectrophotometrically. The DPPH scavenging effect was calculated using the equation:

 $$\begin{array}{c} \text{Scavenging effect}\%=\frac{\left(\text{A}0-\text{A}1\right)}{\text{A}0}\times 100 \end{array}$$

*A*0 (Control Absorbance): *A*0 specifically refers to the absorbance reading of the control solution and A1 (Sample Absorbance): *A*1 represents the absorbance readings obtained for each dilution or concentration of the sample being tested. The IC50, or half-maximal inhibitory concentration, is the concentration of the tested sample required to reduce 50% of the DPPH radicals. IC50 values are determined either graphically or calculated through linear regression analysis of plotted graphs based on inhibition percentages at various concentrations of the tested extracts.

#### 2.6.2. FRAP

The FRAP of the essential oils and extracts was determined according to the Chu, Chang, and Hsu [19] method. This method is based on the reduction of ferric iron (Fe^3+^) to ferrous iron salt (Fe^2+^) by antioxidants that give blue. In this test, 200 *μ*l of examined extracts at different concentrations were mixed with 500 *μ*l of phosphate buffer solution (0.2 M, pH 6.6) and 500 *μ*l of 1% (w/v) potassium ferricyanide solution (K_3_Fe). The contents were incubated at 50°C for 20 min; thereafter, 500 *μ*l of 10% (w/v) trichloroacetic acid was added to stop the reaction, and the tubes were centrifuged for 10 min. Then, 500 *μ*l of the reaction mixture was combined with 500 *μ*l of distilled water and 100 *μ*l of 0.1% FeCl_3_. The absorbance was measured at 700 nm and compared to quercetin, which was used as a positive control. The EC_50_ was calculated from the graph of absorbance at 700 nm against the extract concentration.

### 2.7. Determination of Antimicrobial Activity

#### 2.7.1. Microbial Strains and Growth Conditions

The antibacterial activity was conducted against four strains, as follows: two gram-negative bacteria, *Pseudomonas aeruginosa* (CIP A22) and *Escherichia coli* (CIP 54.8); two gram-positive bacteria, *Enterococcus hirae* (CIP 5855) and *Staphylococcus aureus* (CIP 53154). All bacteria were obtained from the Pasteur Institute. They were maintained by periodic subcultures and preserved at 4°C before use. The tested bacteria were subcultured in the Mueller–Hinton broth for approximately 24 h, followed by 18 h at 37°C, and then adjusted by their absorbance at 600 nm to a dilution between 10^5^ and 10^6^ CFU.

#### 2.7.2. Antimicrobial Test Assay by the Agar Diffusion Method

The antimicrobial assay was detected first by the agar diffusion method described by Hajji et al. [20]. In this well-known procedure, the specific culture medium for each microorganism (Mueller–Hinton) was cast in Petri dishes under well-studied sterility conditions until they solidified. Then, 100 *μ*L of each suspension (10^7^ CFU bacteria/ml) was seeded on the culture medium surface using a sterile flue brush. After that, sterile cellulose discs of 6 mm diameter were impregnated with 50 mg/mL *O. dillenii* extracts and essential oils. Ethanol (70%) was used as a negative control, while ticarcillin, fusidic acid, minocycline, amoxicillin, piperacillin, and rifampicin were used as positive controls. The inoculated plates were incubated at 37°C for 24 h. We evaluated the antibacterial activity by measuring the diameter of the inhibition zone developed around the paper disc in mm, including the diameter of the disc at 6 mm. We carried out the test for three replications, and the resulting values were the averages of three replicates.

#### 2.7.3. Microdilution Method

The minimum inhibitory concentration (MIC) was determined by observing the concentration of the extract that inhibited bacterial growth, indicated by the absence of color change or visible growth after the incubation period. This assay helps in determining the effectiveness of the extract in inhibiting bacterial growth and provides valuable information regarding its antimicrobial properties. The MIC was determined according to Barchiesi et al. [21]. This method was determined using the microdilution method. The first step corresponded to the refilling of each well with 100 *μ*l of Mueller–Hinton and then the extract solution from the second well of the first line. The first column is reserved for the negative control without inoculum, while the last column is reserved for the growth control without extract. We added 10 *μ*l of inoculum to 100 *μ*l of extract and antibiotic at different concentrations in each well. After incubation at 37°C for 24 to 48 h, the results obtained were estimated by visual reading using a developer, which indicates the presence of bacteria. After the incubation period, visual evaluation involved detecting color changes induced by bacterial growth. If wells showed a color change toward pink, it indicated the presence of bacteria. However, wells where the color remained unchanged, staying consistent from the start, signaled the absence of bacterial growth. The lowest concentration of the extract that showed no color change (no bacterial growth) was recorded as the MIC.

### 2.8. Statistical Analysis

Statistical analysis was conducted using ANOVA followed by Tukey's test. The significance of the difference between treatments was considered at *p* < 0.05. and the resulting values were the averages of three replicates. Data are presented as the mean ± standard deviation of the mean. Statistical analyses were performed using SPSS software.

## 3. Results and Discussion

### 3.1. Flower Extract Yields

The choice of solvent has a significant impact on the efficiency of extraction and the properties of the extracts. In fact, each specific combination of plant material and solvent exhibits unique behavior [22]. This is why different solvent options were examined for the extraction of *O. dillenii* flowers, including ethanol-water (70%), ethyl acetate, dichloromethane, butanol, and hexane, using the maceration method. The yields of various extracts obtained through maceration are presented in Table 1 and range from 0.030% (dichloromethane extract) to 19.3% (ethanol 70% extract). Ammar et al. [23] reported yield variations for different extracts of *Opuntia ficus-indica*, ranging from 2.8% (hexane extract) to 14.8% (methanol extract) when using the Soxhlet extraction method and from 2.2% (hexane extract) to 29.7% (water extract) for the maceration method. These yield variations are primarily attributed to the different polarities of the solvents used for extraction. In fact, the 70% ethanol extract has the highest polarity, while dichloromethane and hexane extractions, which have lower polarities, result in the lowest yields. The results suggest that the 70% ethanol extract is particularly effective, likely due to the high dissolving capacity of water. Water, owing to its high polarity, has a strong affinity for the polar compounds in the solute, facilitating their dissolution. The maceration method also allows for easy penetration of the solvent into active sites within the matrix, accelerating the release of extractable compounds [22].

### 3.2. Nutritional Quality

The nutritional quality varied significantly among the studied extracts. The results of the chemical properties of *O. dillenii* are presented in Table 2. The results show that the pH value was 6.32, which was higher than the pH value reported by Ayadi et al. [24] for the spiny cladodes of O. ficus indica (4.02% ± 0.4). The data indicate that *O. dillenii* flowers had a low level of acidity (0.078 ± 0.004%, as citric acid) compared to the acidity of *O. dillenii* pulp (1.42%, as citric acid) and the pH value (3.36), as reported by Embaby et al. [25].

The moisture content of the flowers (74.18%) was significantly lower than that reported by Touil et al. [26] for *O. dillenii* fruits, which were found to be rich in water content (89.6%). The results also indicated that the flower extract had a moderate sugar content (4.27% ± 0.240) and protein content (1.913% ± 0.268). The results demonstrate that the flowers contain 2.267% potassium, making it the dominant macroelement, followed by sodium at 0.55% and calcium at 0.424%. Pavithra et al. [27] reported that *Opuntia dillenii* fruit peel contained 2.12% potassium content, 0.026% calcium, and 0.96% sodium. Moreover, the cladodes, which are parts of cacti consumed as vegetables, contain several essential minerals, among which potassium and calcium are prominent. The content of these minerals typically ranges from about 235 to 5520 mg/100 g of fresh weight. These values showcase the variability in mineral content found in cladodes, highlighting their potential contribution to a balanced diet due to their rich mineral profile, particularly in potassium and calcium [23, 28]. The mineral content depends on the origin of the fruits and the factors of the cultivation site. An accurate understanding of the complexity of the studied environment is essential. The differences in mineral content may be due to interactions between these minerals. Important interactions to consider are the effects of potassium on calcium and sodium, as well as the effects of calcium on sodium.

### 3.3. Chemical Composition of the Essential Oils

Essential oils represent a minor portion of *Opuntia spp.* composition. The chemical compositions of essential oils extracted from *Opuntia dillenii* are presented in Table 3. Moosazadeh et al. [29] identified 19 compounds in essential oils extracted from *Opuntia stricta* fruits, with thymol and n-octane being the major compounds. Bergaoui et al. [30] discovered that *Opuntia lindheimeri* leaves contained a rich composition of esters and carboxylic acids, with esters prevailing in the volatile extracts of the flowers. Butyl tetradecanoate and hexadecanoic acid were the main compounds found in the volatile extract from the flowers. Another study by Wright and Setzer [31] found that the oil derived from *Opuntia littoralis* primarily consisted of compounds from terpenes and fatty acids, where cis-linalool oxide and *trans*-linalool oxide were the main compounds identified. *Opuntia prolifera* oil was predominantly composed of alkanes, with heptadecane as the major compound. Kiralan et al. [32] reported that Tunisian *Opuntia ficus indica* flowers contained a low level of essential oil (0.01%), with benzenacetatealdehyde, D-3-carene, hexanol, and *α*-pinene as the major volatile components.

### 3.4. Chemical Composition of Flower Extracts

#### 3.4.1. Total Phenolic and Flavonoid Contents

**Figure 1:**
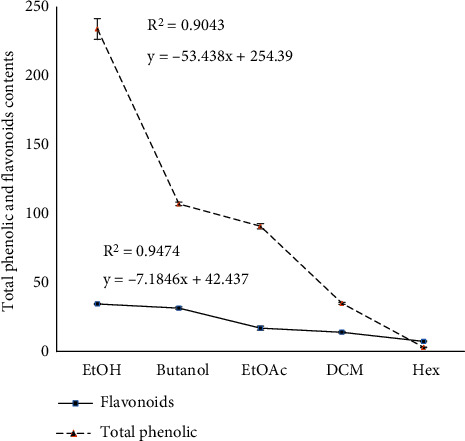
Total phenolic and flavonoid contents in flower extracts of *O. dillenii* (EtOH, ethanol; EtOAc, ethyl acetate; DCM, dichloromethane and Hex, hexane).

The quantities of flavonoids and polyphenols are presented in Figure 1. The total phenolic content ranged from 1.61 ± 0.37 mg GAE/g (hexane extract) to 34.45 ± 0.42 mg GAE/g (ethanol extract). Overall, the total phenolic content of the ethanol extract was found to be higher compared to the other extracts. The total polyphenol content was influenced by the solubility capacity of matrix components, the solvent used during extraction, and the polarity of a hydroethanolic mixture [33]. There were significant differences observed between the extracts, with the highest values of polyphenols and flavonoids obtained in the ethanol extract (234.16 ± 7.63 mg EAG/g and 34.49 ± 0.417 mg equivalent rutin/g, respectively), followed by the butanol extract (107.16 ± 1.18 mg GAE/g and 31.55 ± 0.85 mg equivalent rutin/g, respectively). Ammar et al. [23] reported significant phenolic content in both decoction and infusion, with values ranging from 20.6 to 35.1 mg GAE/100 ml, respectively. On the other hand, Berrabah et al. [34] reported lower values of total phenolic (10.89 ± 5.60 mg/g GAE) and flavonoid (0.96 ± 0.33 mg/g QE) contents in the methanolic extracts of OFI flowers compared to our results. When comparing the total phenolic content in flower extracts with other parts of the plant, it has been reported that the highest concentrations of flavonoids and total phenols are found in the butanol and ethyl acetate extracts. In contrast, the hexane extract contains a very low quantity of phenolic compounds in the peels and cladodes of *O. dillenii* (2.6 and 4 mg/g rutin equivalent per gram of extract, respectively [35].

#### 3.4.2. HPLC Analysis (HPLC–MS)

**Figure 2:**
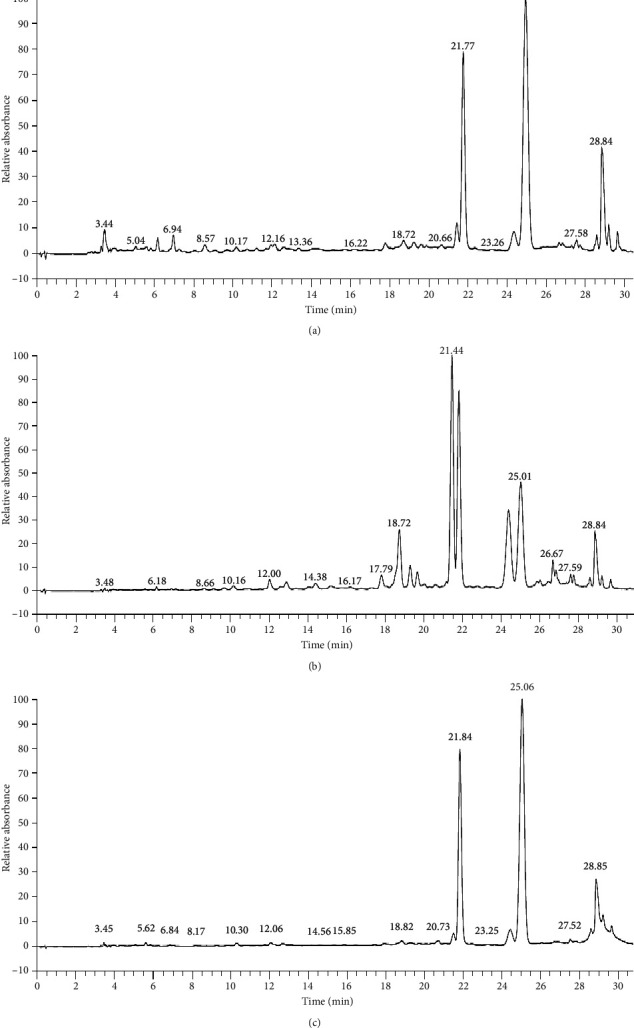
Profile UHPLC-DAD-ESI/MS of (a) ethanol extract, (b) ethyl acetate extract, and (c) butanol extract of *Opuntia dillenii* flowers.

Phenolic compounds were identified based on their retention time, maximum absorbance wavelengths, deprotonated molecules ([M-H]-) in negative ionization mode, and characteristic product ions in comparison with literature data.  Table 4 recapitulates the phenolic compounds identified in extracts from the flowers of *Opuntia dillenii*. The phenolic constituents of the ethanol and butanol extracts of the different parts were detected and identified using UHPLC-DAD-ESI and MS analyses (Figure 2). These compounds were numbered according to their order of elution and are recapitulated in Table 4. The major phenolic compounds found in the ethanol and butanol extracts of the different parts were flavonoids.

The results revealed the presence of secondary metabolites belonging to the class of flavonol glycosides in the *O. dillenii* flowers. The full mass spectra of peaks 1–6 showed deprotonated molecules [M-H] at m/z 609, 477, 623, 301, and 529, respectively. The main compounds identified were glycosides carrying sugar units, specifically quercetin 3-O-rutinoside, isorhamnetin 3-O-glucoside, and isorhamnetin 3-O-rutinoside. In the flowers of *O. dillenii,* the major compounds 4, 5, and 6 showed maximum UV absorption at 254 and 355 nm. Moreover, these compounds displayed [M-H] ions at *m*/*z* = 609, 477, and 623. As a result, they were characterized as quercetin-3-O-rutinoside, isorhamnetin-3-O-glucoside, and isorhamnetin-3-O-rutinoside, respectively. These compounds were detected in the ethanol extract (ODF_Et_) and butanol extract (ODFbut) of the flowers. Nevertheless, a study by Leo et al. [36] in the methanol extract of *O. ficus-indica* showed that the flavonoid compounds that have been identified in extracts of *Opuntia ficus indiça* belong to the class of flavonol glycosides.

Compound 2 has been identified as a quercetin glycoside. The [M-H] ion at *m*/*z* 609 with a fragment at *m*/*z* 463.2 was identified as quercetin 3-O-rutinoside. The [M-H]-*m*/*z* 463 ion (UV max at 255 nm and 354 nm) has been identified as quercetin 3-O-glucoside. Another group of flavonols detected was the isorhamnetin derivatives according to their UV and mass spectra (MS/MS product ion at *m*/*z* 315) as indicated in the studies by Amrane-Abider et al. [6]

Compounds 3 and 4 release [M-H] ions at *m*/*z* 477 and *m*/*z* 623, respectively, and MS fragments at *m*/*z* 315 were identified as isorhamnetin-3-O-glucoside and isorhamnetin-3-O-rutinoside, respectively, as indicated in the studies by Leo et al. [36]. Compound 4 exhibited molecular ions at *m*/*z* 623 and an MS fragment ion at *m*/*z* 315, which corresponded to the loss of a rhamnosyl glucoside fragment, which was consistent with the presence of a rutinoside fragment and allowed its identification as isorhamnetin 3-O-rutinoside as indicated in the studies by El-Hawary et al. [7].

Compound 5 (Rt = 26.67 min) showed an [M-H]-ion at *m*/*z* 301 in the UV spectrum (255 and 355 nm), which made it possible to identify it as quercetin. Flavonoids, particularly the derivatives of quercetin and isorhamnetin, were the main phenolic compounds found in flowers. Isorhamnetin-3-O-rutinoside was the main flavonoid identified, representing values of 47.08% and 52.54% in the ethanol and butanol extracts, respectively. It was followed by quercetin-3-O-rutinoside, which represented values of 25.59% and 30.36%, respectively, while isorhamnetine 3-O-glucoside showed values of 2.87% and 2.86%, respectively. Our results agree with previous reports on the genus *Opuntia* and with studies on *O. ficus-indica* fruits [37, 38]. A study conducted by Ahmed et al. [3] reported the identification of three isolated compounds from flowers, isorhamnetin-3-O-*β*-D-rutinoside, kaempferol 3-O-*α*-arabinoside, and isorhamnetin-3-O-*β*-D-glucopyranoside. Additionally, these molecules have been noted for a wide array of important biological and therapeutic properties, particularly antibacterial and anti-inflammatory effects [5] (Table 4). Additionally, Ouerghemmi et al. [39] reported that ferulic acid and quercetin were found in higher concentrations than other phenolic compounds in *Opuntia ficus-indica* flowers. Aruwa, Amoo, and Kudanga [40] reported that the compounds present in the flowers of *Opuntia ficus indica* are gallic acid, 7-isorhamnetin 3-O-galactoside, and 6-isorhamnetin 3-O-robinobioside. Certainly, Leo et al. [36] identified kaempferol 3-O-arabinoside, quercetin 3-O-rutinoside, and isorhamnetin 3-O-glucoside in methanol extracts of *Opuntia ficus-indica* flowers from Italy. Concerning cladodes, some reports have indicated that quinic acid and myricetin were the most found compounds in *Opuntia dillenii* cladodes [41]. However, Missaoui et al. [42] reported a higher abundance of piscidic acid and isorhamnetin derivatives in *O. ficus-indica*.

#### 3.4.3. Identification of Isolated Compounds and NMR Spectroscopy

UHPLC-DAD-ESI/MS analysis of the butanol extract (ODFbut) showed the presence of a major peak. The butanol extract was purified using column chromatography, and the product obtained was analyzed by mass spectrometry and NMR (Table 5). The analyses make it possible to identify a compound with a mass of 623.2 m/z. This compound corresponds, according to the literature, to a flavonoid: isorhamnetin-3-O-rutinoside.

The compound was obtained as a yellow powder. The ESI/MS showed an [M-H] ion at *m*/*z* = 623.2. The MS and ^13^C NMR data indicated a molecular formula of C_28_H_32_O_16_. The ^1^H NMR spectrum showed two doublets at **δ**^1^H = 6.253 (d, *J* = 2.0 Hz, 1 H) and **δ**^1^**H** = 6.477 (d, *J* = 2.0 Hz, 1 H), which correlated with the carbon signals at **δ**^13^**C** = 98.81 and 93.83. A doublet of doublets at **δ**^1^**H** = 7.58 (dd, *J* = 8.4, 2.0 Hz) and two doublets at **δ**^1^**H** = 6.976 (*J* = 8.4 Hz) and 7.912 (*J* = 2.0 Hz) were assigned as C6′-H, C5′-H, and C2′-H. Additionally, the ^1^H NMR spectrum exhibited one singlet at **δ**^1^**H** = 3.89 (3 H), indicating the presence of a methoxy group (OCH_3)_, which showed a correlation with the carbon resonance at **δ** *C* = 146.8 (C3′). The doublets at **δ** *H* = 5.49 (d, *J* = 7.5 Hz) and **δ** *H* = 4.47 were attributed to the anomeric protons of the glucosyl and rhamnosyl moieties, respectively. The analysis of the ^1^H and ^13^C NMR spectra of the compound, along with a comparison with values from the literature by Boubaker et al. [43], confirmed the compound's identity as isorhamnetin-3-O-rutinoside. Additionally, Amrane-Abider et al. [6] conducted a molecular analysis of biochemical compounds extracted from *Opuntia ficus-indica* flowers, where they identified the presence of quercetin, quercetin-3-O-rutinoside and isorhamnetin 3-O-rutinoside.

### 3.5. Antioxidant Activity

The antioxidant activity of essential oils and flower extracts of *O. dillenii* was evaluated by DPPH using a spectrophotometer following the reduction of this radical, which is accompanied by its change from violet (DPPH•) to yellow (DPPH-H). A phytochemical study of *O. dillenii* extracts from flowers showed significant levels of polyphenols and flavonoids. Reactive oxygen species (ORS) have a great ability to damage almost all types of cellular constituents in the body, which explains their involvement in the induction and/or amplification of several pathologies. The supplementation of the body by exogenous antioxidants is very useful to fight against these harmful species. The DPPH radical is recovered by the antioxidant compounds present in the extracts by proton donation to form reduced DPPH, which can be quantified by its decrease in absorbance [44]. The flowers of *Opuntia dillenii* have been found to contain natural phenolic compounds, including quercetin, isorhamnetin, isorhamnetin 3-O-rutinoside, and quercetin-3-O-rutinoside. This suggests that they could be a source of antioxidants [6]. The evaluation of the antioxidant activity of the different extracts showed antioxidant power with respect to free radicals, which confirms the results of the determination of flavonoids and polyphenols. The antioxidant capacity was proportional to the extract concentration, which was evaluated by determining the EC50 that corresponds to the concentration of an antioxidant needed to decrease the initial concentration of DPPH by 50%. Evaluation of the antioxidant potency of the DPPH extracts showed variations in the DPPH trapping activity in extracts from different parts of *O. dillenii*. In comparison to the standard antioxidant (quercetin), the extracts had a lower level of antioxidant activity, with an EC of 4.5 *μ*g/mL. This antioxidant activity may be due to phenolic compounds, which are recognized as potentially antioxidant substances with the ability to trap radical species and reactive forms of oxygen.

**Figure 3:**
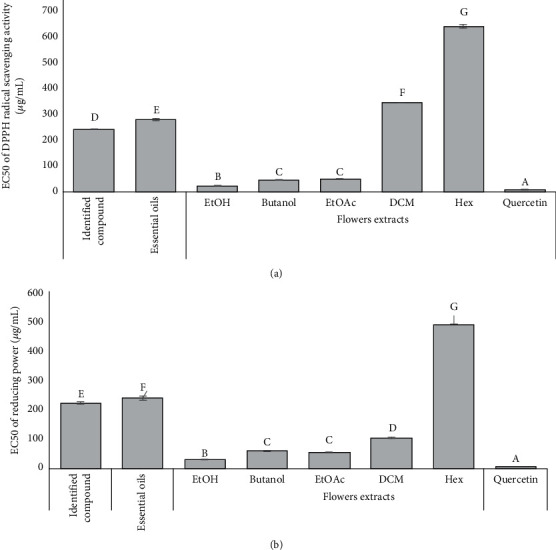
EC50 values of antioxidant activities of *O. dillenii* flower extracts; (a) DPPH radical scavenging activity; (b) reducing power (ETOH, ethanol; EtOAc, ethyl acetate; DCM, dichloromethane; Hex, hexane).

The results of the antioxidant activity exerted on the free radical DPPH by extracts of the flowers of *O. dillenii* are expressed by the parameter EC50 and are shown in Figure 3. The extract with the lowest EC50 value exerts the most potent antifree radical activity. The extracts have antioxidant activity, which varies from extract to extract for different or the same plant sample. The comparison between the extracts shows that the ethyl acetate and butanol extracts exhibit more effective DPPH radical scavenging capacities than the crude extracts (ethanol). This explains why most of the antioxidant substances were entrained by the two organic solvents during the fractional process. While the lowest overall activity is seen in hexane extracts, Ammar et al. [23] reported that the methanol (MeOH) extracts from *Opuntia ficus-indica* exhibited an antioxidant activity with flowers displaying an EC50 value of 100 ± 7.2 *μ*g/mL. Similarly, Alimi et al. [45] also reported on the antioxidant activity of *O. ficus-indica* using MeOH extracts, where the flowers exhibited an EC50 value of 140 *μ*g/mL. The antioxidant activity of *O. ficus-indica* has been extensively studied [46].

The reducing power of iron (Fe3+) in the extract was assessed following the methodology described by Oyaizu [47]. This method allows the estimation of the reducing capacity of substances within the extract by measuring their ability to convert Fe^3+^ to Fe^2+^ and quantifying this alteration by observing the resulting color change. An increase in absorbance indicates an elevation in the activity of reducing power. The findings revealed that the extracts acted as electron donors and could effectively reduce Fe^3+^ ions in a concentration-dependent manner.

As depicted in Figure 3, the flower extract exhibited superior Fe^3+^ reduction activity. Notably, the ethyl acetate and butanol extracts displayed significantly different and noteworthy reducing powers compared to the crude extract (ethanol) and most of the dichloromethane and hexane extracts. These observed properties are attributed to the nature of the reducing substances present in the extracts. The results highlight that the ethyl acetate and butanol extracts possess the strongest reducing power among the tested extracts.

### 3.6. Determination of Antimicrobial Activity

The diameters of the inhibition zones (including the diameter of the disc) of different strains with the extracts studied are presented in Table 6. The obtained results show various actions and degrees of microorganism sensitivity toward the tested extracts. Analysis of these results makes it possible to conclude that the flower extracts exhibit marked antibacterial activity against *P. aeruginosa* and *Staphylococcus aureus*. On the other hand, this inhibition remains lower than that of the positive controls*. S. aureus* is sensitive to most of the extracts studied, except for the hexane extract, which has no activity against this strain. The best activity was detected for the ethyl acetate extract of flowers against the strains studied. We have observed that the inhibitory power against the microorganisms tested does not depend on the morphology or on the gram. Indeed, we have shown antibacterial activity against *S. aureus* (Gram +) as well as *P. aeruginosa* (Gram −). The extract shows low activity for *E. coli* and *Enterococcus hirae*. Thus, the present results show that the extracts of the flowers of *Opuntia* are active to varying degrees and show antibacterial activity by inhibiting the growth of bacterial strains. The most effective extract vis-à-vis the bacterial strains studied appears to be the same extract that has significant antioxidant activity, which indicates the concordance of these two biological properties. Evaluation of the antibacterial capacity and determination of the MIC of the extracts of *Opuntia dillenii* by the microdilution plate method revealed that all the extracts showed an inhibitory effect against certain bacterial strains. The most sensitive microorganism was *Staphylococcus aureus*. The highest activity was obtained with the ethyl acetate extract. Following these results, the extracts offered very interesting antibacterial characteristics for the strains. The antimicrobial properties of plant extracts have been attributed to their chemical profiles. Indeed, the antibacterial action of extracts is justified by their richness in phenolic compounds. Several studies have shown that phenolic compounds have high antibacterial power. In addition, the antibacterial activity can be attributed to the synergy between all the constituents extracted from *Opuntia*. The synergistic relations between the different compounds may be at the origin of a more pronounced activity than is foreseeable for the majority of compounds. Due to the presence of active compounds, the extracts were found to be effective against the strains studied. Ammar et al. [2–23] reported the presence of bioactive compounds possessing antimicrobial properties within *Opuntia ficus-indica* flowers. Their study demonstrated the potential of flower extracts in combating microorganisms including, *Staphylococcus aureus, Pseudomonas aeruginosa,* and *Escherichia coli*. These flower extracts exhibit promise for application across diverse sectors such as the phytosanitary, cosmetic, and pharmaceutical industries.

### 3.7. Principal Component Analysis (PCA)

**Figure 4:**
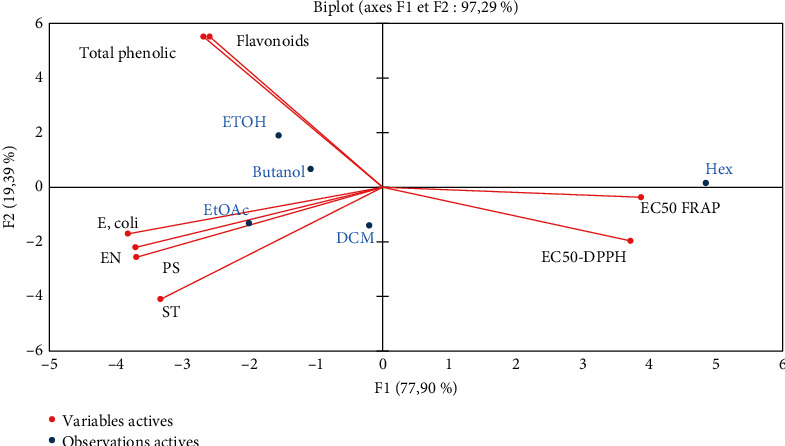
Principal component analysis (PCA) biplot showing the correlation between polyphenol content, flavonoids, antioxidant activity, minimum inhibitory concentration (MIC), and various flower extracts.

The PCA was employed to investigate the correlation between polyphenol content, flavonoids, antioxidant activity, MIC, and different flower extracts (Figure 4). The initial two principal components, F1 (77.9%) and F2 (19.39%), accounted for 97.29% of the overall variation. The results of the PCA unveiled significant levels of phenolic compounds in the flowers, coupled with a strong ability for DPPH radical scavenging. Certainly, the study observed a positive correlation between the concentrations of polyphenols and flavonoids and both the antibacterial and antioxidant activities. This study represents the first comprehensive analysis of the chemical composition of *Opuntia* dillenii flowers cultivated in Morocco, along with an assessment of their antioxidant and antibacterial properties. The flowers exhibited richness in sugar, minerals, proteins, total phenolic, and flavonoid compounds. The antioxidant potential of *O. dillenii* extracts, evaluated through diverse tests such as DPPH radical scavenging and reducing power, displayed variations among the extracts. In summary, this research suggests that *O. dillenii* flowers could serve as valuable sources of antioxidant compounds, highlighting the potential of this species for further exploration regarding its benefits and potential applications within the *Opuntia* genus.

## 4. Conclusion

This study focused on evaluating extracts obtained from *Opuntia dillenii* flowers as sources of natural antioxidants. Based on the HPLC data obtained in this study, it was found that *Opuntia dillenii* flowers are a source of natural phenolic compounds such as isorhamnetin 3-O-rutinoside and quercetin-3-O-rutinoside. These results suggest that *Opuntia dillenii* flowers should be considered a source of antioxidants with significant antioxidant and antimicrobial activity. The analysis revealed a positive relationship between phenolic and flavonoid contents and antioxidant and antibacterial activity. These findings emphasize the importance of further research and exploration of *Opuntia dillenii*, as it appears to have valuable benefits and potential applications. *Opuntia dillenii* is an *Opuntia* species that deserves increased attention in terms of studies and research.

## Figures and Tables

**Table 1:** Extraction yield of *Opuntia dillenii* flower extracts prepared by the maceration method (EtOH, ethanol; EtOAc, ethyl acetate; DCM, dichloromethane; Hex, hexane).

**Extract**	**E**_**t**_ **OH 70%**	**Butanol**	**E**_**t**_ **OAc**	**DCM**	**Hex**
Extract yield (%)	19,344^a^ ± 1	5.5^b^ ± 0.5	1.04^c^ ± 0.15	0.3^d^ ± 0.02	1.7^c^ ± 0.2

*Note:* The letters indicate statistically significant differences between the groups, with each letter representing a distinct group as per the results of the analysis.

**Table 2:** Chemical composition of *Opuntia dillenii* flowers.

**Parameter**	**Contents %**
Water	74.18
Ashes	9.31 ± 0.68
Acidity	0.078 ± 0.004
Sugar	4.27 ± 0.240
Protein	1.913 ± 0.268
Na^+^	0.550 ± 0.085
Ca^2+^	0.424 ± 0.061
K^+^	2.267 ± 0.178
pH	6.32

**Table 3:** Composition of essential oils from *Opuntia dillenii* flowers.

**No**	**Name**	**RT**	**Formula**	**Content %**
1	Hemellitol	9 539	C_9_H_12_	0.86
2	decane	9 903	C_10_H_22_	0.81
3	Mésitylène	10 722	C_9_H_12_	0.91
4	M-cymene	12 454	C_10_H_14_	1.39
5	2-Methylpropylbenzene	12 789	C_10_H_14_	0.99
6	4-Ethyl-o-xylene	13 294	C_10_H_14_	0.89
7	p-Cymene	13 417	C_10_H_14_	1.19
8	4-Ethyl-1,2-dimethylbenzene	13 710	C_10_H_14_	0.98
9	Undecane	14 637	C_11_H_24_	3.75
10	Dodecane	19 450	C_12_H_26_	1.10
11	Coumaran	20 085	C_8_H_8_O	1.51
12	Citronellol	20 632	C_10_H_20_O	3.93
13	Geraniol	21 705	C_10_H_18_O	1.11
14	3′-Methoxy-acetophenone	24 296	C_9_H_10_O_2_	7.56
15	Dodecamethylcyclohexasiloxane	24 398	C_12_H_36_O_6_Si_6_	1.38
16	Cyclomethicone 7	31 532	C_14_H_42_O_7_Si_7_	1.25
17	2-Phenylethyl tiglate	35 737	C_13_H_16_O_2_	1.11
18	Hexadecane	36 608	C_16_H_34_	3.76
19	Gamma-Eudesmol	37 175	C_15_H_26_O	5.18
20	6,9-Heptadecadiene	39 128	C_17_H_32_	2.21
21	Octadecane	43 909	C_18_H_38_	5.45
22	Trimethylsilyl myristate	45 551	C_17_H_36_O_2_Si	0.86
23	Hexadecanoique acid	49 236	C_16_H_32_O_2_	2.04
24	Eicosane	50 538	C_20_H_42_	5.42
25	Trimethylsilyl hexadecanoate	51947	C_19_H_40_O_2_Si	12.79
26	Docosane	56 602	C_22_H_46_	4.01
27	Hexacosane	59 445	C_26_H_54_	1.49
28	Tetracosane	62 184	C_24_H_50_	3.22
29	Heptadecane	64 815	C_17_H_36_	2.07
30	Bis(2-ethylhexyl) phthalate	65 352	C_24_H_38_O_4_	2.26
31	9-Octylheptadecane	68 440	C_25_H_52_	3.32
32	Heneicosane	69 636	C_21_H_44_	1.91
33	Squalene	69 721	C_30_H_50_	4.59
34	Tetracosane	70 650	C_24_H_50_	3.83
35	Triacontane	71 565	C_30_H_62_	1.24

**Table 4:** UHPLC-DAD-ESI/MS of ethanol (ETOH), ethyl acetate (EtOAc), and butanol extract of *Opuntia dillenii* flowers.

**No**	**Rt**	**(M-H)- (m/z)**	**UHPLC max (nm)**	**Identified compounds**	**Flowers extract %**		
					**EtOH**	**EtOAc**	**Butanol**
1	18.72	303	295	Taxifolin	—	11.86	—
2	21.84	609	255, 355	Quercetin 3-O-rutinoside	25.59	23.42	30.36
3	24.37	477	255, 355	Isorhamnetin 3-O-glucoside	2.87	12.89	2.86
4	25.06	623.2	254, 356	Isorhamnetin-3-O-rutinoside	47.08	17.37	52.54
5	26.67	301	255, 355	Quercetin	0.55	1.8	0.28
6	27.19	315	255, 357	Isorhamnetin	—	3.76	—
7	28.84	529	261	1-Caffeoyl-5-feruloylquinic acid	13.16	5.96	1.72

**Table 5:** Nuclear magnetic resonance spectral data of isorhamnetin 3-O-rutinoside.

**Chemical shifts of isorhamnetin 3-O-rutinoside, ** ^**1**^ **H, ** ^**13**^ **C**				
**N**	***δ*** ^**1**^ **H**	***δ*** ^**1**^ **H [43]**	***δ*** ^**13**^ **C**	***δ*** ^**13**^ **C [43]**
2	—	—	149.41	156.3
3	—	—	133	133
4	—	—	177.27	177.3
5	—	—	161.17	161.2
6	6.253	6.2 (1H, d, *J* = 2.0)	98.81	98.7
7			nd	164.2
8	6.477	6.4 (1H, d, *J* = 2.0)	93.83	93.8
9	—	—	156.48	156.4
10	—	—	103.86	104
1′	—	—	121.04	121
2′	7.912	8.0 (1H, d, *J* = 1.8)	113.28	113.4
3′	—	—	146.8	147
4′	—	—	nd	143
5′	6.976	6.9 (1H, d, *J* = 8.2)	115.24	115
6′	7.58	7.5 (1H, dd, *J* = 8.5, 1.8)	122.25	122
OCH_3_	3.89	3.85 (s)	55.65	55.9
5-OH	12.618	12.6	—	—
7-OH	nd	9.8	—	—
1^″^	5.49	5.4	103.86	101.8
2^″^	3.468	3.56 (m)	70.2	71.1
3^″^	3.4	3.41 (m)	74.28	73
4^″^	—	—	70.2	70.4
5^″^	3.77	3.59 (m)	nd	73.5
6^″^	3.32 –3.77	3.30–3.60	66.82	65.1
1^‴^	4.47	4.42	101.21	100
2^‴^	—	—	68.2	68.3
3^‴^	3.28	3.28	70.58	70.6
4^‴^	3.1	3.06	71.78	71.9
5^‴^	nd	3.61	68.27	68
6^‴^	1.03	1.04 (d)	17.68	17.9
OH	4.47, 5.13, 5.49	4.56, 4.82, 5.2, 5.4	—	—

**Table 6:** Evaluation of the antibacterial activity of *Opuntia dillenii* extract and essential oil: inhibition diameter in mm and minimum inhibitory concentration (MIC).

	***Escherichia coli***		***Staphylococcus aureus***		***Pseudomonas aeruginosa***		***Enterococcus hirae***	
	**Diameter in mm**	***MIC*(mg/ml)**	**Diameter in (mm)**	***MIC*(mg/ml)**	**Diameter in mm**	***MIC*(mg/ml)**	**Diameter in mm**	***MIC*(mg/ml)**
Identified compound.	10 ± 0.9	6.25	9 ± 0.5	6.25	10.33 ± 1.5	6.25	9.00 ± 1.00	6.25
Essential oil	9 ± 0.8	6.25	10 ± 1.5	6.25	11 ± 1.5	6.25	9.5 ± 0.5	6.25
Flowers								
EtOH	8.50 ± 0.9	6.25	10.33 ± 1.5	6.25	11.33 ± 1.5	6.25	9.00 ± 1	6.25
Butanol	8.00 ± 1.00	6.25	11.00 ± 1.00	6.25	12.00 ± 1	6.25	9.00 ± 1	3.125
EtOAc	11.33 ± 0.6	3.125	15.33 ± 0.6	6.25	14.00 ± 1	3.12	11.50 ± 0.5	3.125
DCM	8.33 ± 1.2	6.25	12.83 ± 0.3	12.5	12.00 ± 1	12.5	10.83 ± 0.8	6.25
Hex	ND	ND	ND	ND	7 ± 0.3	ND	6 ± 0.5	ND
Antibiotic								
TIM	15 ± 00	ND	18 ± 00	ND	16 ± 00	ND	21 ± 1	ND
Fosfomycine	9 ± 00	ND	8 ± 03	ND	8.5 ± 0.3	ND	10 ± 0.5	ND
FD	10 ± 0	ND	8 ± 0.5	ND	7.5 ± 0.5	ND	7 ± 0.1	ND
AMC	10 ± 0	ND	9 ± 0.3	ND	9 ± 0.2	ND	11 ± 0.5	ND
MH	14 ± 0.3	ND	15 ± 0.4	ND	14 ± 0.3	ND	15 ± 0.7	ND
RD	13 ± 0.42	ND	12 ± 0.35	ND	11.5 ± 0.5	ND	14 ± 0.3	ND
TZP	15 ± 1	ND	22 ± 1.2	ND	20 ± 1	ND	25 ± 1.5	ND
Amoxicillin	ND	0.062	ND	0.062	ND	ND	ND	0.031

Abbreviations: AMC, amoxicillin; DCM, dichloromethane; EtOAc, ethyl acetate; ETOH, ethanol; FD, fusidic acid; Hex, hexane; MH, minocycline; RD, rifampicin; TIM, ticarcillin; TZP, piperacillin.

## Data Availability

The data used to substantiate the findings of this study are contained in the article. Nevertheless, additional information required can be obtained from the corresponding author upon request.
